# Supplementary figures and images for: The protein phosphatase PPM1A dephosphorylates and activates YAP to govern mammalian intestinal and liver regeneration
Source: PLoS Biol. 2021 Feb 25;19(2):e3001122. doi: 10.1371/journal.pbio.3001122 (PMC7978383; doi:10.1371/journal.pbio.3001122)

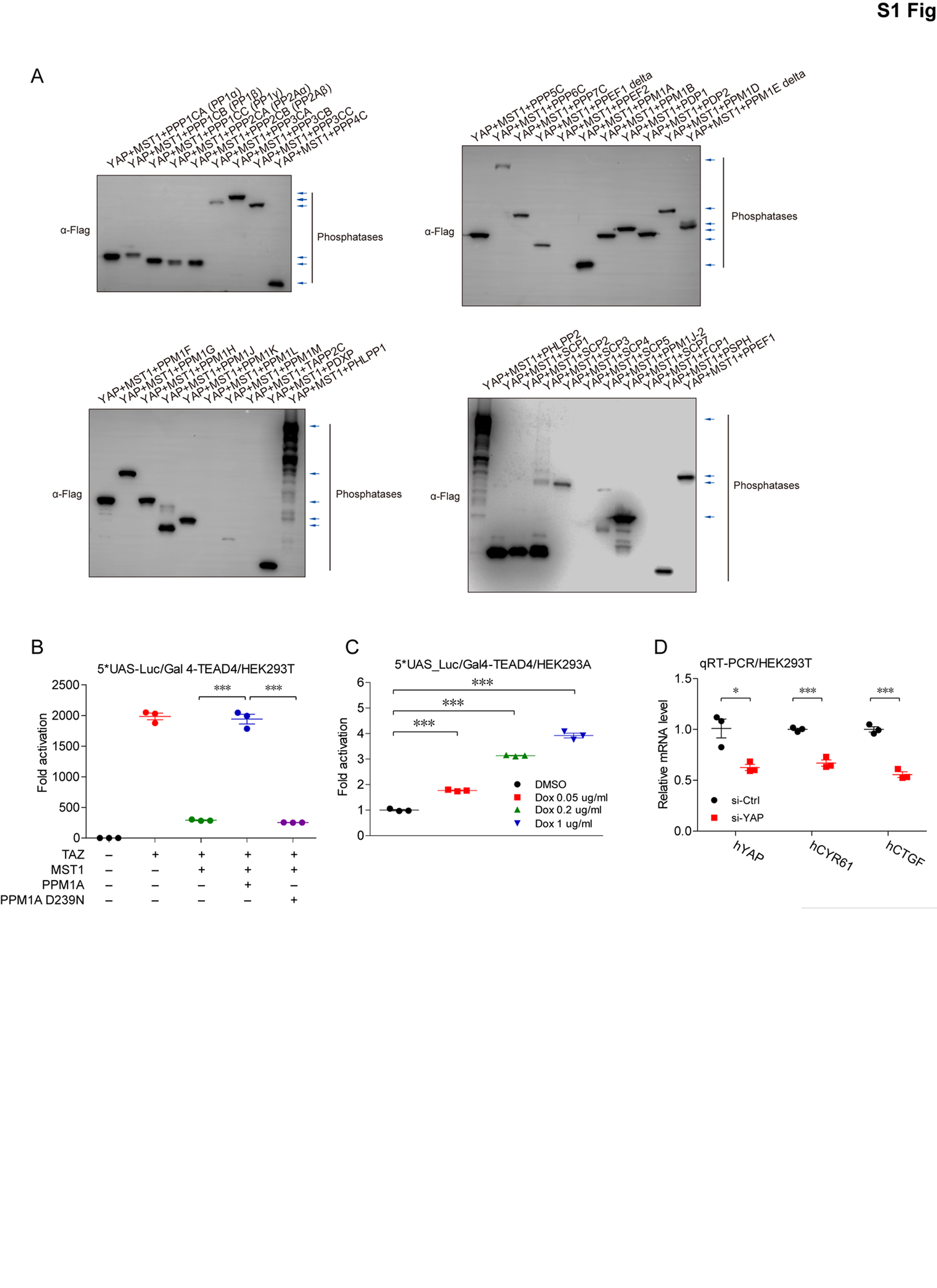

Supplement: S1 Fig — (A) The protein levels of individual phosphatases used in the phosphatome screening was shown by the immunoblotting targeting their Flag tags. (B) Transcription potency of TAZ, which was suppressed by coexpression of MST1, was profoundly recovered by cotransfection of wild-type PPM1A but not its phosphatase-dead form (D239N). (C) The activity of TEAD-responsive promoter was enhanced in the inducible expression of PPM1A in a dose-dependent manner. (D) Depletion of YAP down-regulated the mRNA levels of CYR61 and CTGF, an indication to validate them as target genes of the YAP/TAZ-TEADs complex. Unprocessed images of blots are shown in S1 Raw Images. Statistics source data are provided in S1 Data. MST1, mammalian sterile 20-like kinase 1; PPM1A, protein phosphatase magnesium-dependent 1A; TAZ, transcriptional coactivator with PDZ-binding motif; TEAD, transcriptional enhanced associate domain; YAP, Yes-associated protein. (TIF) [file pbio.3001122.s001.tif]

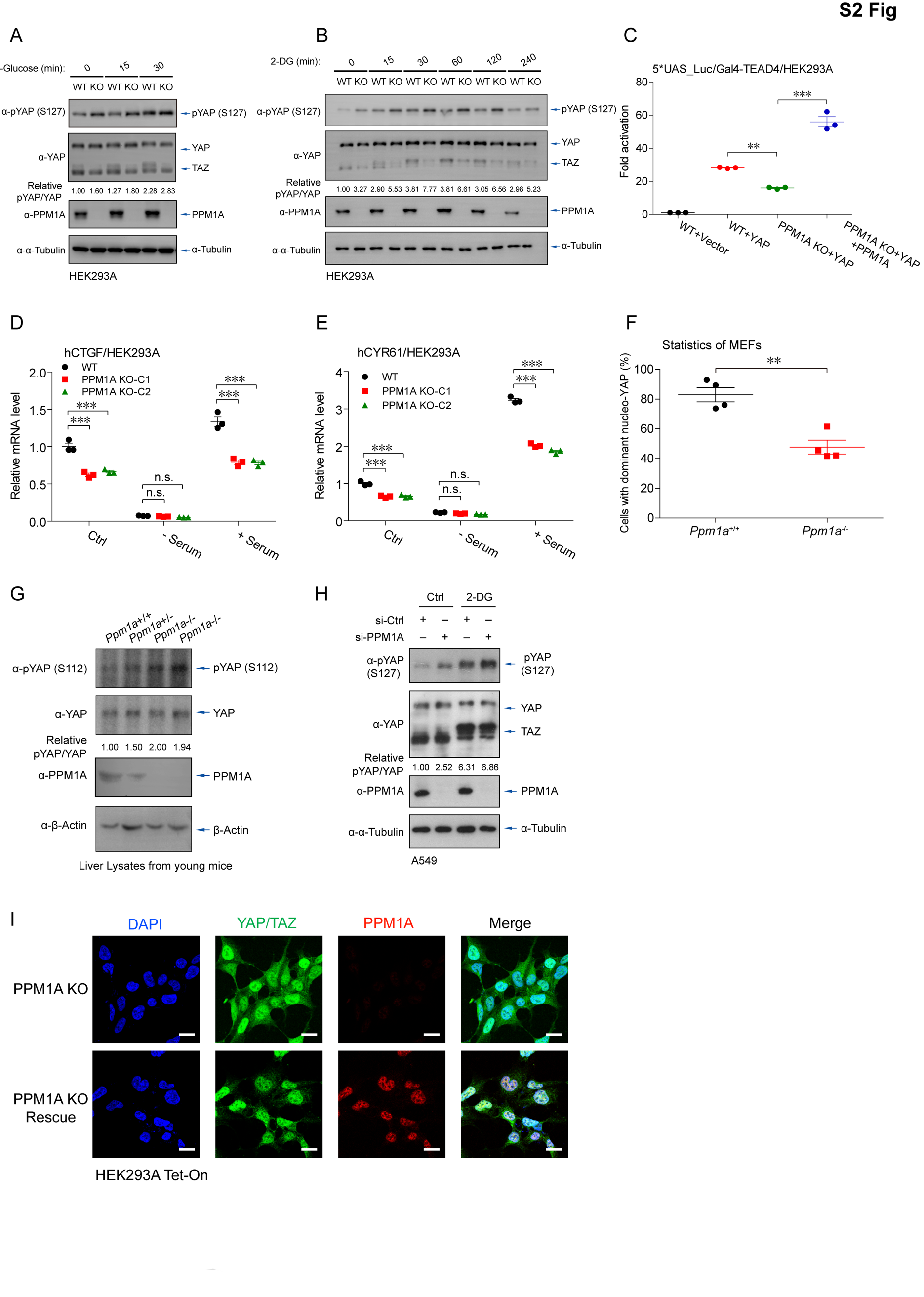

Supplement: S2 Fig — (A) Glucose starvation and PPM1A KO similarly triggered the up-regulation of phospho-YAP (S127). (B) Genetic ablation of PPM1A in HEK293 cells resulted in an enhanced level of phospho-YAP (S127) and an exaggerated TAZ degradation, in a degree similar to cells with energy deficiency. (C) Reintroduction of ectopic PPM1A in PPM1A KO HEK293 cells restored the suppressed activity of the YAP/TAZ-TEAD promoter. (D, E) The decreased mRNA levels of CTGF (D) and CYR61 (E) were detected by qRT-PCR assays in the absence of endogenous PPM1A. (F) A statistics for cells with the dominant nucleo-YAP in MEFs from WT and PPM1A KO mice. (G) An enhanced level of phospho-YAP (S112) was detected in the lysates of livers of young homozygous PPM1A KO mice. (H) PPM1A depletion in A549 cells by siRNA interference resulted in an increased phosphorylation level of YAP at the S127 residue. (I) Reconstitution of PPM1A restored the nuclear distribution of endogenous YAP/TAZ in PPM1A KO cells. Unprocessed images of blots are shown in S1 Raw Images. Statistics source data are provided in S1 Data. KO, knockout; MEF, mouse embryonic fibroblast; phospho-YAP, phosphorylating forms of YAP; PPM1A, protein phosphatase magnesium-dependent 1A; qRT-PCR, quantitative real-time PCR; siRNA, small interfering RNA; TAZ, transcriptional coactivator with PDZ-binding motif; TEAD, transcriptional enhanced associate domain; WT, wild-type; YAP, Yes-associated protein. (TIF) [file pbio.3001122.s002.tif]

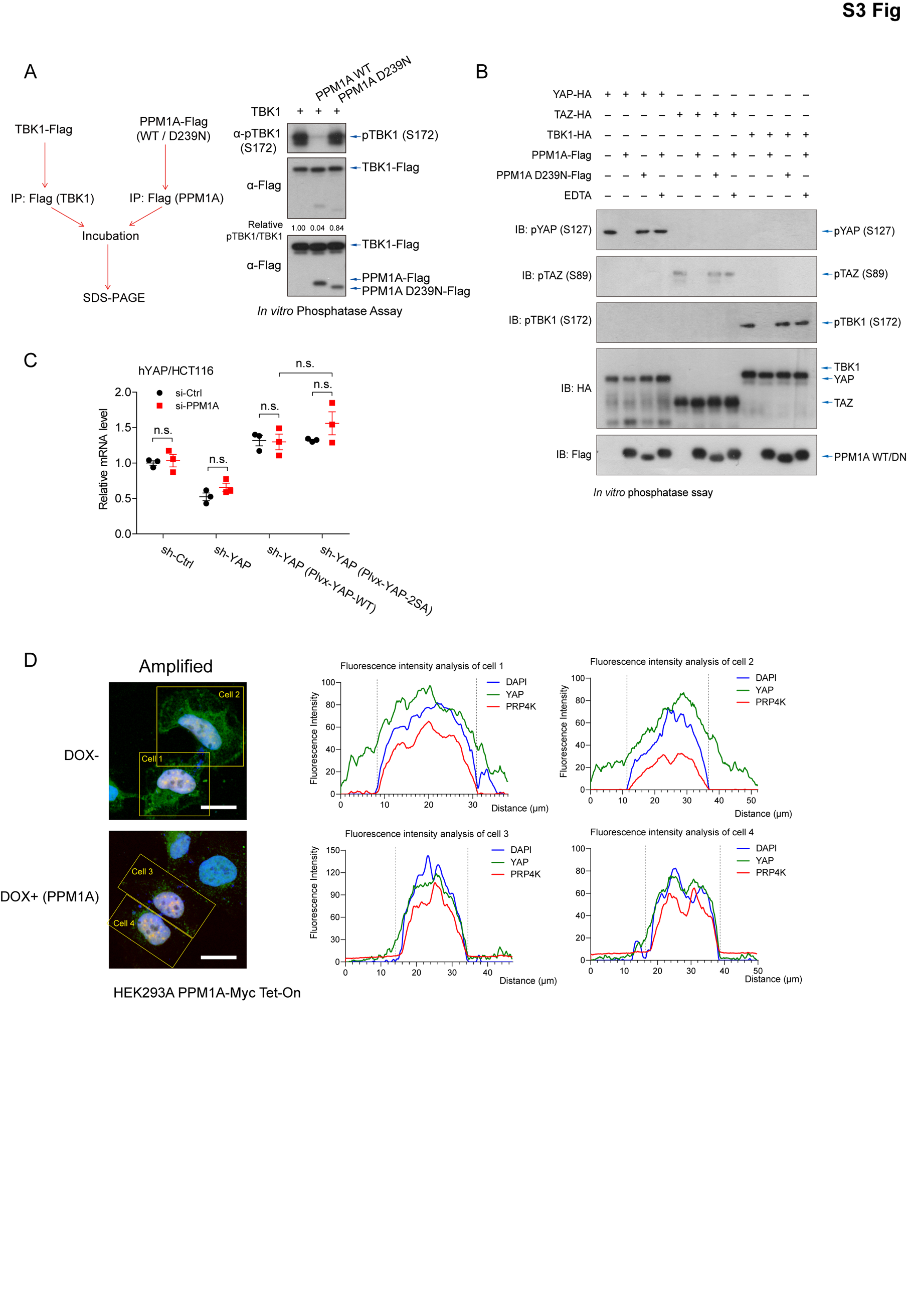

Supplement: S3 Fig — (A) PPM1A purified from cells was active, as evidenced by its capability to dephosphorylate TBK1, a substrate previously reported [40]. (B) Purified PPM1A dephosphorylated YAP, TAZ, and TBK1 during an in vitro phosphatase assay, which required enzymatic activity of PPM1A and Mg2+/Mn2+. (C) An indication for the successful reconstitution of YAP WT and 2SA mutant in YAP-depleted HCT116 cells. (D) Fluorescence intensity analysis by Image J software indicated the obvious presence of cytoplasmic-YAP in PRP4K-expressed cells, which was diminished by PPM1A induction. Unprocessed images of blots are shown in S1 Raw Images. Statistics source data are provided in S1 Data. PPM1A, protein phosphatase magnesium-dependent 1A; TAZ, transcriptional coactivator with PDZ-binding motif; TBK1, TANK-binding kinase 1; WT, wild type; YAP, Yes-associated protein. (TIF) [file pbio.3001122.s003.tif]

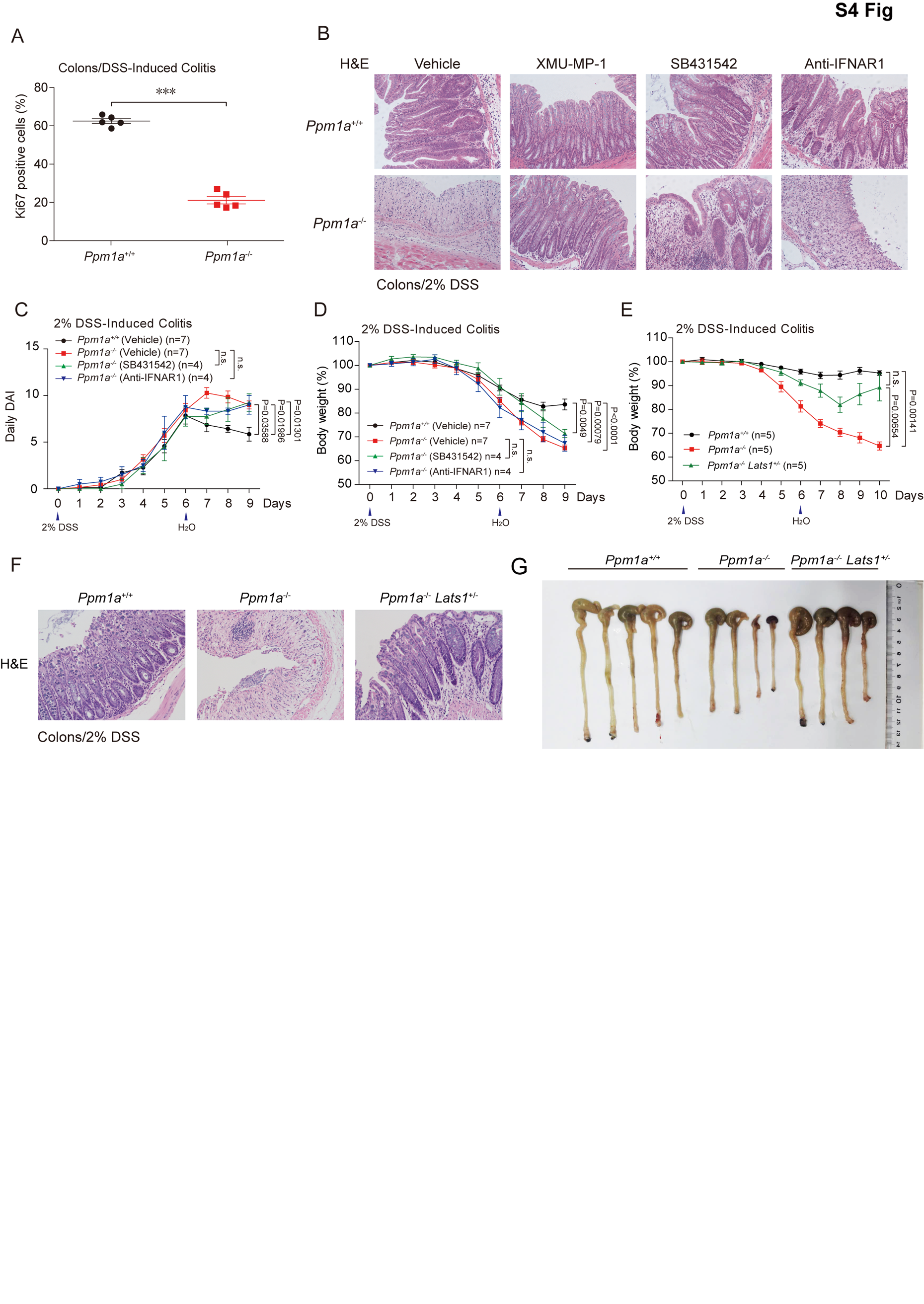

Supplement: S4 Fig — (A) Decrease of proliferating cells (Ki67 positive) of Ppm1a−/− intestinal epithelium was calculated. (B) Administration of the MST1/2 inhibitor XMU-MP-1 largely prevented DSS-induced disruption of crypts and villus architectures in PPM1A KO mice. (C, D) Pharmacological blockade of TGF-β signaling by SB431542, or IFN-I signaling by anti-IFNAR1 neutralizing antibody, failed to protect the DSS-induced colitis. (E–G), Genetic deficiency of LATS1 (Lats1+/−) survived PPM1A KO mice from the DSS-induced colitis attack (E), largely preserved the villus structure of Ppm1a−/− intestines (F), and partially recovered the colon length (G). Unprocessed images of blots are shown in S1 Raw Images. Statistics source data are provided in S1 Data. DSS, dextran sulphate sodium; IFN-I, type I interferon; KO, knockout; LATS1, large tumor suppressor kinase 1; MST1/2, mammalian sterile 20-like kinase 1 and 2; PPM1A, protein phosphatase magnesium-dependent 1A; TGF-β, transforming growth factor beta. (TIF) [file pbio.3001122.s004.tif]
