# Supplementary material for: The protein phosphatase PPM1A dephosphorylates and activates YAP to govern mammalian intestinal and liver regeneration
Source: PLoS Biol. 2021 Feb 25;19(2):e3001122. doi: 10.1371/journal.pbio.3001122 (PMC7978383; doi:10.1371/journal.pbio.3001122)

S1 Raw Image

Figure 1

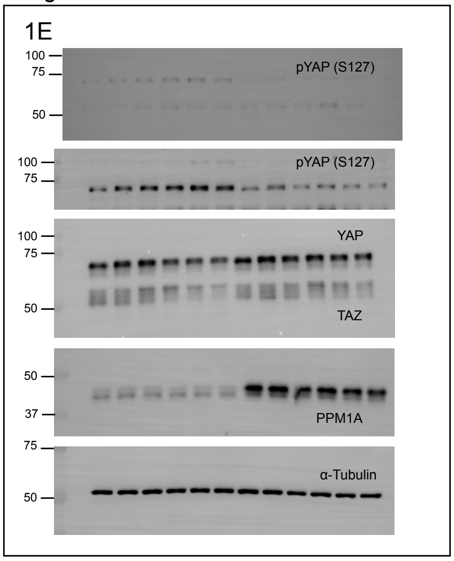

Figure 1

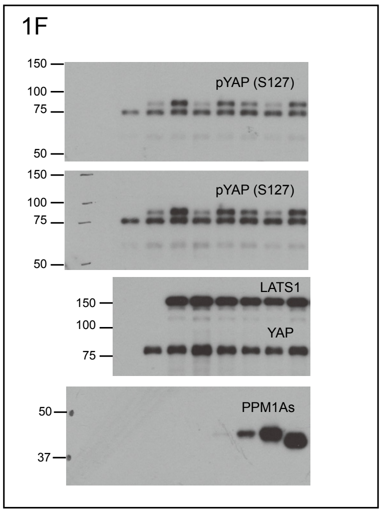

Figure 1

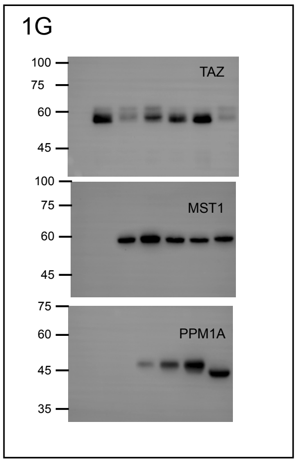

Figure 1

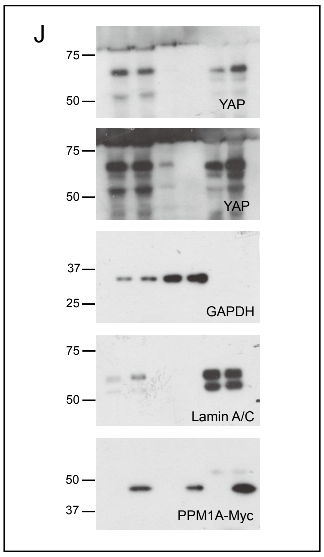

Figure S1

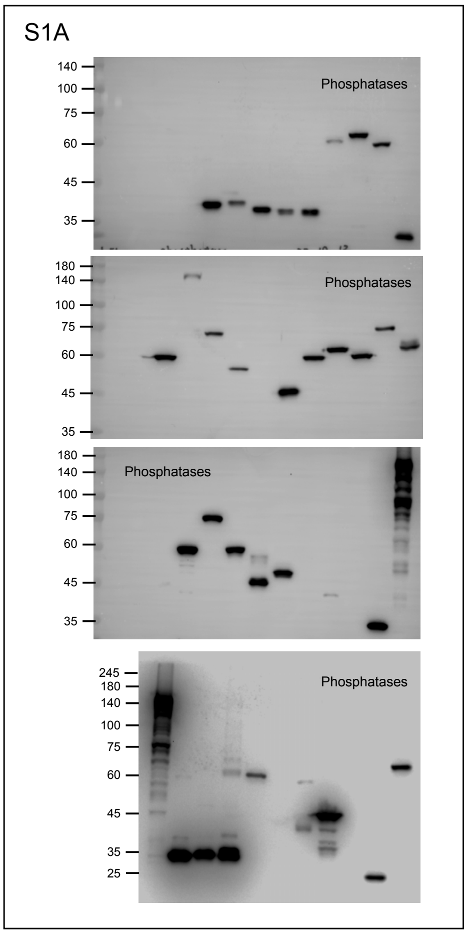

Figure 2

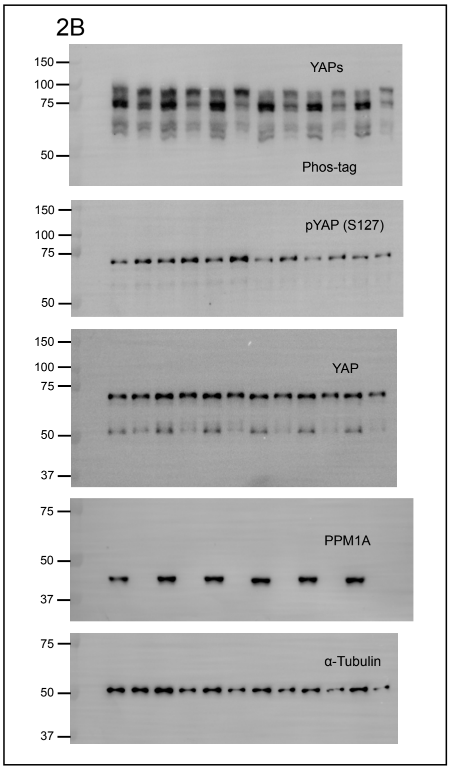

Figure 2

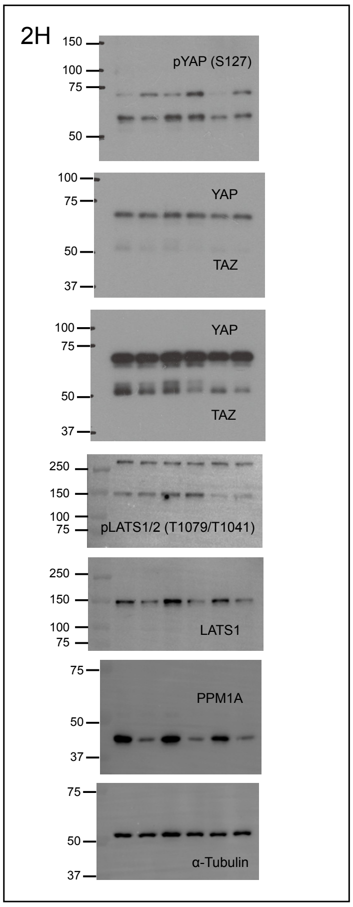

Figure 2

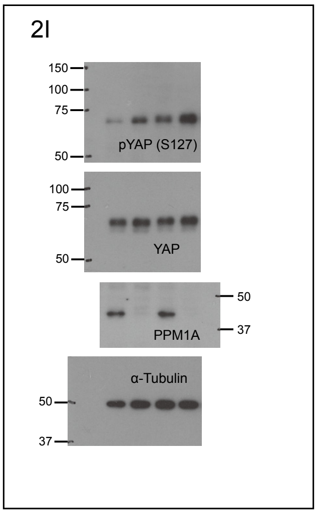

Figure 2

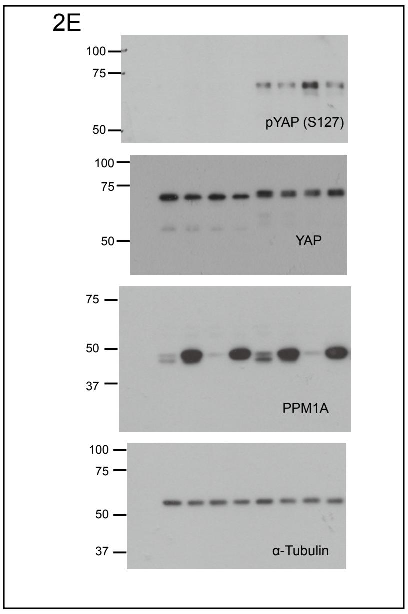

S1 Raw Image

Figure S2

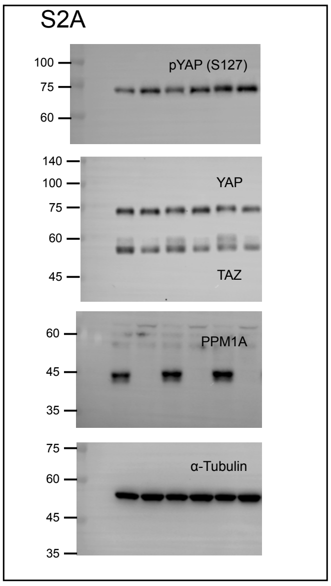

Figure S2

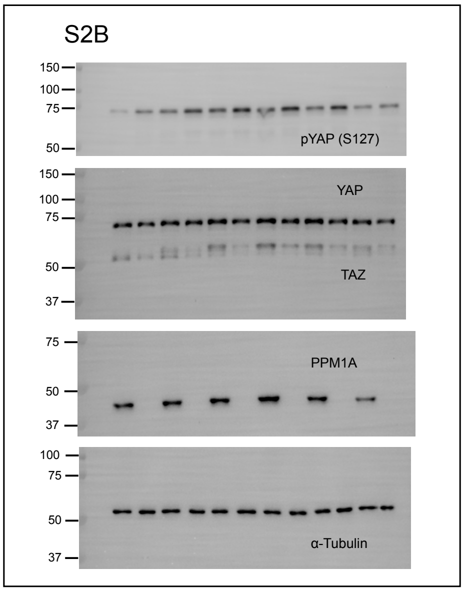

Figure S2

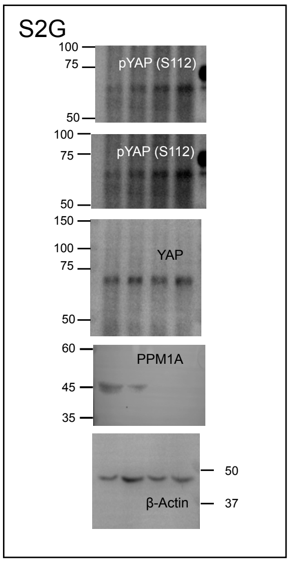

Figure S2

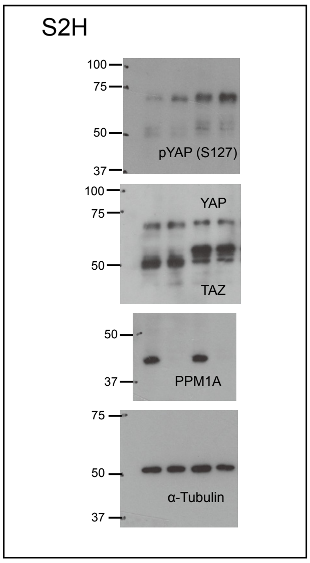

Figure 3

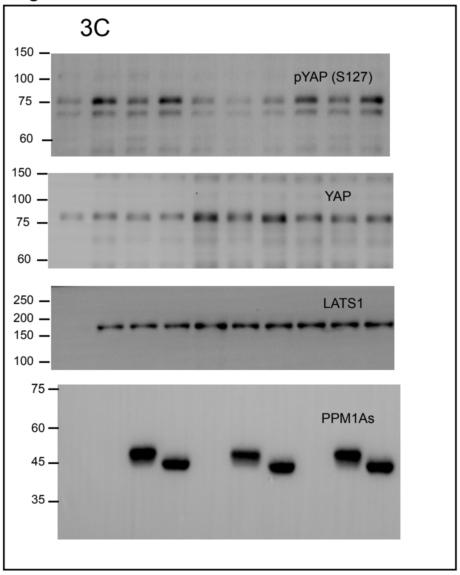

Figure 3

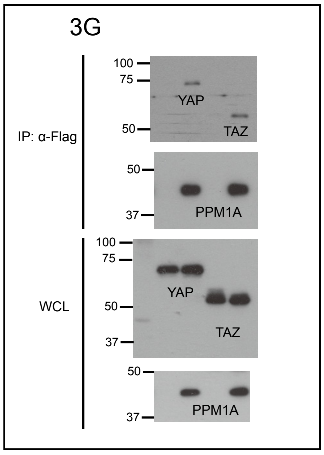

Figure 3

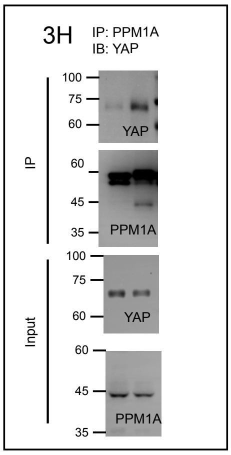

Figure 3

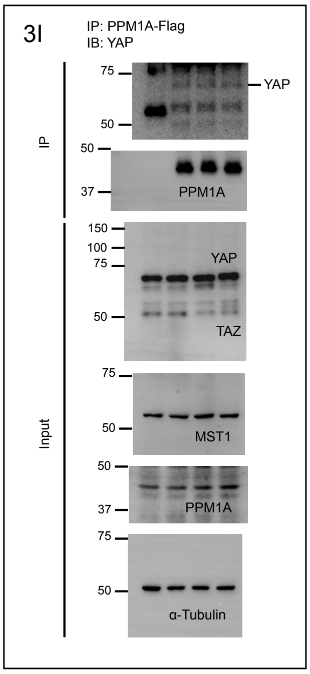

Figure 3

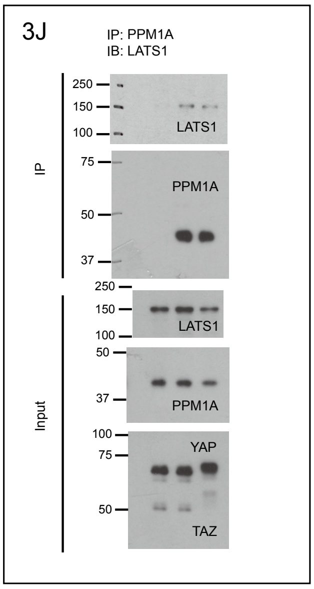

Figure 4

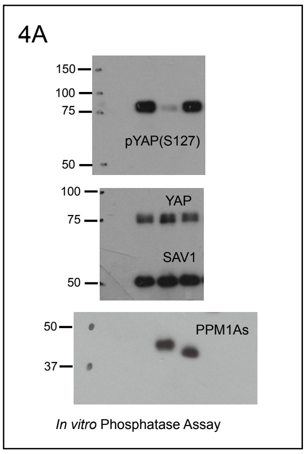

Figure 4

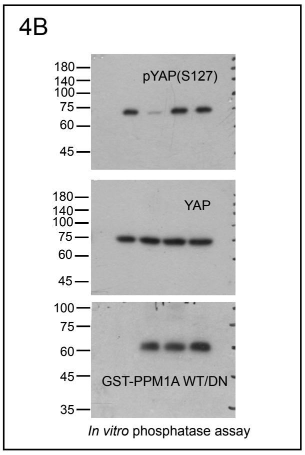

Figure 4

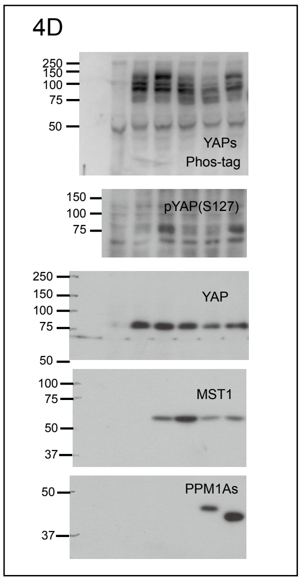

S1 Raw Image

Figure S3

S3A

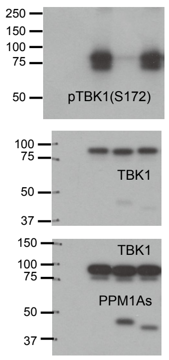

In vitro Phosphatase Assay

Figure S3

S3B

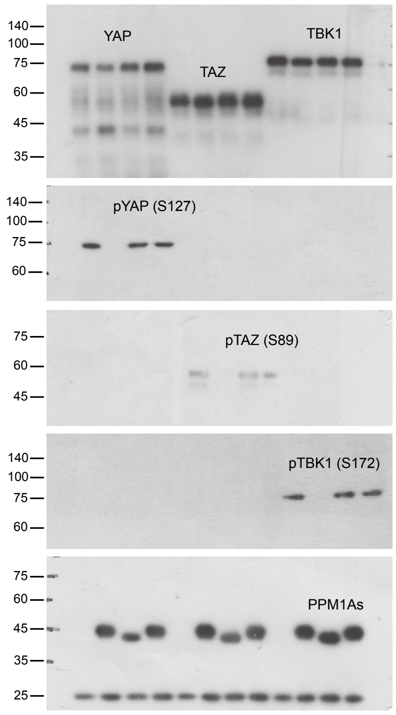

In vitro phosphatase assay

Figure 5

5A

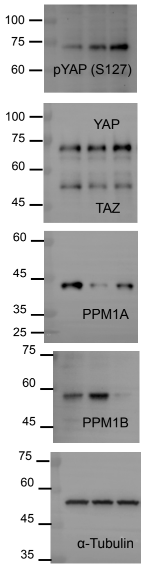

Figure 7

7H

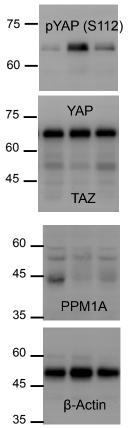

Supplement: S1 Raw Images — (PDF) [file pbio.3001122.s010.pdf]
