# Supplementary material for: The protein phosphatase PPM1A dephosphorylates and activates YAP to govern mammalian intestinal and liver regeneration
Source: PLoS Biol. 2021 Feb 25;19(2):e3001122. doi: 10.1371/journal.pbio.3001122 (PMC7978383; doi:10.1371/journal.pbio.3001122)
Supplement: Attachment — Submitted filename: Response to revewers_PBIOLOGY-D-20-01910R1.pdf [file pbio.3001122.s011.pdf]

Response to the reviewers' comments:

We thank the reviewers for their efforts in evaluating our manuscript and their positive and very constructive comments. We have performed serials of experiments to address all the reviewers' concerns.

In particular, we provide additional evidence, including (1), In vitro phosphatase assays with recombinant PPM1A from the bacteria and measurement of the PPM1A enzymatic kinetic coefficient with a YAP peptide, both convincingly suggest that YAP is a substrate of PPM1A. (2), The fact that function of phosphorylation-resistant version of YAP (YAP S109A/S127A) is barely affected by PPM1A in cells and organoids. (3), Genetic rescue of the severe colitis phenotypes in PPM1A KO mice by crossing with the Lats1$^{+/-}$ mice. (4), The results that PPM1B assists and compensates PPM1A to jointly regulate YAP phosphorylation, which explains the minimal development defects in PPM1A KO mice. (5), Evidence that both the phosphorylation and activity of TAZ are directly regulated by PPM1A. (6), The interaction between YAP and PPM1A in tissues at the endogenous level. (7), Evidence for the enhanced phospho-YAP (S127) level in tissue sections and lysates upon PPM1A deletion. (8), The fact that pharmacological inhibition of MST1 in liver generation model restores the defects in PPM1A KO mice. In addition, we have strengthened the proposed mechanism and manuscript with various improved controls and data qualities, revised the manuscript text with necessary corrections and clearer description.

These new or updated data are shown in Figs.1E, 1G, 1J, 2H, 3B, 3C, 3H, 4B, 4C, 4F, 5A, 5B, 5F, 6A, 6B, 6F, 6H, 6I, 7B, 7F, 7G. and suppl. Figs. S1A, S1B, S1D, S2A, S2F, S2G, S2I, S4A-S4D, S6C-S6G. We believe that after these tremendous efforts, especially with the enzymatic kinetics measurement and the genetic rescue of colitis phenotypes by Lats1$^{+/-}$ mice, we have adequately addressed all concerns/questions raised by the reviewers, and the revised manuscript is significantly improved and suitable for publication in PLoS Biology. Below are our point-by-point responses to the reviewers' comments:

`

Remarks to the Author:

Rev. 1:

In this manuscript, the authors report the identification of PPM1A as a new regulator of YAP function. Zhou et al. use a phosphatome screen to identify putative phosphatases that regulate YAP function and identify PPM1A, a metal-dependent phosphatase as a hit in their screen. PPM1A expression counteracts MST-, LATS- and MAP4K-dependent phosphorylation of YAP at Ser127, one of the critical residues that regulates YAP function and that is involved in the control of YAP nuclear translocation. The authors show that PPM1A expression promotes YAP nuclear localization and correlates with increased YAP target gene expression. The authors also suggest that loss of PPM1A function by CRISPR is associated with increased YAP cytoplasmic retention. PPM1A KO intestinal organoids have proliferation defects and, in vivo, PPM1 KO mice have deficient gut and liver regeneration upon

injury. The authors suggest that the in vivo defects of PPM1A KO animals are related to the regulation of YAP and not to the regulation of other pathways where PPM1A has shown to be involved, such as the TGF-beta or IFN pathways.

This report addresses an important question in the Hippo pathway field, regarding the regulation of YAP phosphorylation by phosphatases. So far, it is still unclear whether there are specific phosphatases that directly regulate specific YAP phosphorylation events. However, despite the fact that the manuscript tackles an important point of regulation within the Hippo pathway, there are particular issues with the manuscript that prevent its publication in its current form. Below are points to be addressed by the authors.

Major points:
The main criticism of the manuscript relates to the fact that the authors suggest that PPM1A directly targets YAP and dephosphorylates it. However, in my opinion, the data supporting this conclusion is not robust enough.

1. The authors fail to address in the manuscript whether the effect of PPM1A on YAP phosphorylation is indeed direct or due to changes in the phosphorylation of upstream components (e.g. MST1). This is an important point to address as it relates to the mechanistic effect of PPM1A in the pathway. Since the in vitro phosphatase assay is not performed with recombinant PPM1A but with PPM1A immunoprecipitated from cells, it is possible that the effect is indirect and not due to PPM1A itself. Although the authors show an in vitro kinase assay where PPM1A supposedly dephosphorylates YAP on Ser127, Fig. 4A lacks a positive control to ensure that PPM1A purified from cells is active. Ideally, a known target of PPM1A should be tested in the same conditions.

Thanks for the reviewer's positive comments and suggestions. Per reviewer's advice, in the revised manuscript we have further clarified the direct dephosphorylation of YAP by PPM1A. First, we performed in vitro phosphatase assays with recombinant PPM1A from the bacteria. Clearly, GST tagged recombinant PPM1A from E.coli effectively eliminated the YAP phosphorylation at the S127 residue, while phosphatase-dead PPM1A and metal-deprived PPM1A not (Fig. 4B). As expected, purified PPM1A from cells also dephosphorylated TBK1, a positive control in consistent with previous report [1] (Fig. S4A, S4B). Furthermore, using the malachite green assay, we measured the enzymatic reaction kinetic coefficient of PPM1A against the YAP peptide spanning S127 residue (sequence of QHVRAH-pS-SPASLQ). The retrieve of a robust specificity constant of K_{cat}/K_m, convincingly indicates that YAP is a substrate of PPM1A (Fig. 4C). In addition, we validated that function of phosphorylation-resistant version of YAP (YAP 2SA, the S109A/S127A mutant) was barely affected by PPM1A (Fig. 4F, S4C, 5H), which we will elaborate in detail in the next question. We believe that these consistent observations, in addition to evidence in the previous manuscript such as the result that PPM1A restored the

PRP4K-mediated YAP suppression, can convincingly establish a point that PPM1A directly dephosphorylate YAP. We have added these new data and accordingly revised the text in the manuscript.

2. Moreover, the MS data provided by the authors shows that LATS is detected in the control condition but not when PPM1A is added to the reactions. Why would this be the case? And could this account for the differences in YAP phosphorylation levels? If LATS remained in a complex with YAP in one condition but not the other, this could obviously affect the extent of phosphorylation that occurs and the final levels of p-YAP in the in vitro kinase assay. Related to this point, data shown in Fig. 2H regarding the levels of p-LATS should be quantified. Does PPM1A affect the levels of p-LATS in Thr1079? If so, is the effect of PPM1A on YAP phosphorylation actually an indirect effect of controlling LATS phosphorylation?

Thanks for the reviewer's insightful observations and suggestions. Please first allow us to give a detailed description of the mass spectrometry analysis we performed. The mass spectrometry samples were sourced from the in vitro phosphatase assay, including separately expressed and purified PPM1A and phospho-YAP, which was cotransfected with MST1 kinase and SAV1 scaffold protein SAV1. Samples were separated by SDS-PAGE, stained by coomassie blue to locate YAP, and collected by a cut spanning YAP (length and width are roughly about 1cm). Because the experiment did not take all proteins in the samples and the cutting would not be exactly the same, the results of the interacting proteins from this experiment were biased. In addition, it is understandable that the YAP-LATS1 interaction was altered when another YAP-interacting molecule was added to the system with a large amount.

Furthermore, we showed a very intriguing interaction between LATS1 and PPM1A. PPM1A was triggered to translocate into the cytoplasm by LATS1 or Hippo signaling activation, where it co-localized with LATS1 there (Fig. 3D-3F). Therefore, it is possible the interaction between PPM1A and LATS1 may alter the LATS1-YAP complex. Furthermore and as we have suggested in previous manuscript, PPM1A may also dephosphorylate and regulate the components of the LATS-YAP complex, including LATS1. As revealed in Fig. 2H, which we quantified the pYAP (S127) and pLATS1 (T1079) levels, PPM1A deficiency indeed resulted in a lower level of total LATS1, although depletion of PPM1A did not significantly change the level of phosphorylated LATS1. However, the presence of similar pLATS1 levels in comparison to a dramatic enhanced levels of pYAP may suggest that YAP, but not LATS1, is dephosphorylated by PPM1A in vivo.

Given the solid evidence to indicate a direct PPM1A-YAP modification (please referred to last question), we designed two additional experiments to address this puzzling question. First, we observed that PPM1A directly dephosphorylated the LATS1 (T1079D)-mediated phosphorylation of YAP (Fig. 3B-3C), indicating that PPM1A effects on YAP but not LATS1. Second, we introduced into intestinal organoids of the

YAP 2SA mutant (S109A/S127A), which resists to PPM1A (according to immunoblotting and ms data) but not so effective to resist LATS1 (which generally requires a 5SA YAP), entirely restored the molecular phenotypes of on Hippo-YAP signaling in Ppm1a$^{-/-}$ organoids (Fig. 5H). Therefore, these two observations, as well as evidence for the direct PPM1A-YAP modification and PPM1A's regulation on RPR4K-mediated YAP suppression, suggest that PPM1A directly and functionally effects on YAP. However, whether PPM1A modifies and regulates the other components of LATS1 complex, such as the LATS1 and its protein stability, is currently unknown and waiting for further investigation.

3. If PPM1A is indeed essential to regulate YAP Ser127 phosphorylation, one would expect that the KO phenotypes would be more severe and that the mice would not be viable. The authors suggest that PPM1B may compensate for PPM1A as the double KO mice were lethal. Do the authors have any data that supports this argument, either in vitro or in vivo? Are the in vitro phenotypes associated with PPM1A loss of function altered when PPM1B levels are modulated?

Per reviewer's request, we generated HEK293 cells with double depletion of both PPM1A and PPM1B by the siRNA interference, from which we observed a further enhancement of the pYAP (S127) level, when compared to PPM1A-knockdown cells (Fig. 5A). In addition, compensation in protein level between PPM1A and PPM1B was suggested (Fig. 5A). Furthermore, the colony formation, which partially reflected an activity of YAP/TAZ, was severely downregulated when both PPM1A and PPM1B were depleted (Fig. 5B). These observations thus indicate the redundant effects between these two closely-related phosphatases in regulation of Hippo-YAP signaling.

On the other hand, disruption of PPM1B leads to early pre-implantation lethality [2]. The Ppm1b$^{d/d}$ gene-trap mice, as we have reported previously [3], are viable and born with a normal Mendelian distribution. However, no offspring could be successfully obtained of Ppm1b$^{d/d}$/Ppm1a$^{-/-}$ mice, indirectly supporting our hypothesis that PPM1B may compensate for PPM1A during the embryonic development in Ppm1a$^{-/-}$ mice.

4. If the function of PPM1A in the Hippo pathway is indeed to mainly regulate Ser127 phosphorylation, its effects should be dispensable when cells express a non-phosphorylatable version of YAP. This should be tested.

Per the reviewer's advice, we reconstituted WT YAP and its non-phosphorylatable form (YAP 2SA, S109A/S127A) into the YAP-depleted HCT116 cells. A substantial increase in mRNA levels of YAP/TAZ targets was observed in cells with expression of YAP 2SA (Figs. 4F, S4C), suggesting that S127 and S109 are the major residues modified by PPM1A. Furthermore, we similarly introduced intestinal organoids of the YAP 2SA mutant (S109A/S127A), which resists to PPM1A (according to immunoblotting and ms data), entirely restored the molecular phenotypes of PPM1A

KO organoids in Hippo-YAP signaling (Fig. 5H). Therefore, both observations indicated that PPM1A dephosphorylates YAP majorly on S127 and S109, which effects on Hippo-YAP signaling.

5. Lines 394-396: The authors state that PPM1A and YAP dephosphorylation are necessary for regeneration. However, there is no data on YAP Ser127 phosphorylation from in vivo experiments.

Per reviewer's suggestion, we examined the level of pYAP (S112) (equivalent of human S127) in the lysates obtained from the regenerated livers. An obvious increase of pYAP (S112) was seen in Ppm1a$^{-/-}$ livers, which was alleviated by the small molecular MST inhibitor (Fig. 7F). In addition, the substantially increased level of pYAP (S112) was observed in Ppm1a$^{-/-}$ colons by IHC, which was largely restored upon LATS1 deficiency (Fig. 6I). We have added these new data and accordingly revised the text in the manuscript.

6. How was the analysis of the Mass Spectrometry data performed? There is no corresponding Methods section for the MS experiments, no mention of how many times the experiments were performed and whether experiments included technical replicates or not. The data in Suppl Table 1 for Fig. 5 is not clear enough to understand what type of analysis was done. Were the total number of YAP peptides found in the experiments 21 (Control) and 17 (PPM1A) as suggested in Supp Table 5? Can the levels of phosphorylation be compared without a reliable form of quantification? Differences in total protein amount, coverage and peptide detection could have a significant impact in the interpretation of the results. Is there a specific reason why peptides spanning the Ser127 site are not detected in the MS samples?

We apologized for the missing of methodologies for mass spectrometry analysis in the previous manuscript, which now has been complemented. This Nano LC/tandem MS analysis is for protein identification, characterization, and label-free quantification. The samples have been scanned in mass spectrometry for 3 times without technical replicates but only the testing with best quality was used for analysis, as the experimental nature of ms. The coverage of YAP is comparatively poor (50-60%) comparing to most other proteins we examined (usually 70-95% coverage). Because a total of 142 and 152 hits of pan-YAP peptides were respectively detected in control and PPM1A group, we believe the hits amount is adequate enough for a reliable analysis. S127 residue is among a very long YAP peptide (AHsSPASLQLGAVSPGTLTPTGVVSGPAATPTAQHLR, 37 a.a.) due to the lack of proteinase cleave site, which greatly compromised its detection.

7. Is PPM1A specific for YAP or does it also target TAZ? If it is the former, how is PPM1A specifically targeted to YAP?

Per the reviewer's suggestion, we conducted experiments to investigate the effect of

PPM1A on TAZ. We found that PPM1A regulated the activity of TAZ, as indicated by the reporter assay and the observation for Hippo signaling-induced TAZ degradation and phosphorylation at S89 (Figs. 1G, S1B, S4B). Obviously, PPM1A restored the MST1-induced suppression of TAZ-responsive promoter (Fig. S1B), and PPM1A stabilized TAZ in a dose-dependent manner (Fig. 1G). PPM1A also interacted with both YAP/TAZ with a similar affinity (Fig. 3G), and directly dephosphorylated TAZ (sFig. 4B). Therefore, we conclude that PPM1A targets both YAP and TAZ during the regulation of Hippo signaling.

8. With regards to the results from the phosphatome screen (Fig. 1A and 1B) why is there such a big difference between YAP+MST1 in Fig. 1A and Fig. 1B? In Fig. 1B, YAP+MST1 leads to virtually no activation of YAP, whereas in Fig. 1A there is a ~200-fold induction compared to negative controls. If possible, results from the phosphatome screen and related figures should have accompanying data regarding the levels of the relevant proteins in the assay. Could the differences in YAP readout activation be due to changes in protein levels?

Thank you for the note. The difference of YAP+MST1 in Fig. 1A and Fig. 1B is due to a different amount of MST1 expression plasmids was used in two experiments for complying by their distinct purposes. In the experiment of phosphatase screening (Fig. 1A), we employed a very lower amount of MST1 plasmid in transfection (10 ng), to ensure we can sensitively detect even the minor effect of individual phosphatase. In contrast in Fig. 1B, we used a significant higher amount of MST1 plasmid in transfection (50 ng) to maintain a high activation state of Hippo signaling, in order to validate the strong effect of PPM1A on inhibiting the Hippo signaling. We added the corresponding description in legend. In addition, we supplied the immunoblottings for the protein levels for the individual phosphatases in screening (Fig. S1A).

9. Line 230-232: Authors suggest that PPM1A localization is controlled by LATS. How does this correlate to the fact that in Fig. 1J, PPM1A levels seem to be equivalent in the nucleus and cytoplasm in basal conditions?

We quantified Fig. 1J which showed apparently that in the resting state, there was higher level of PPM1A in the nucleus than those in the cytoplasm in HEK293 cells (5th panel), which was similar to those observed by the immunofluorescence imaging in Fig. 1I.

10. In Fig. 2E, why is there a complete absence of p-YAP Ser127 in the absence of 2-DG? In previous experiments under the same conditions (Fig. 1E) there is extensive YAP phosphorylation.

The level of pYAP (S127) in resting state is often between detectable and non-detectable, depending subtly on minor changes of cell state and immunoblotting conditions. This nature of pYAP in resting state is often seen in various literatures. In

the case of Fig. 1E, we can also see an undetectable pYAP in the resting state upon short film exposure. We updated this figure panel.

11. Data related to Fig. 2F should be quantified. Does reconstitution of the KO MEFs rescue YAP subcellular localization?

Per the reviewer's suggestion, a quantitative analysis was made for 8 representing immunofluorescence images of MEFs from WT and Ppm1a$^{-/-}$ mice, which revealed a substantially decreased level of cells with the dominant nucleo-YAP, upon PPM1A deletion (Fig. S2F). We failed to obtain the primary MEFs with PPM1A reconstitution, as their viability was drastically reduced during drug treatment. However, rescue of PPM1A in PPM1A-KO HEK293 cells showed that it drove the translocalization of YAP to the nucleus (Fig. S2I).

12. Lines 227-229: Based on the data shown in Fig. 3D, it is impossible to determine if there is indeed co-localization of PPM1A and YAP, or PPM1A and LATS in the cytoplasm. The readout used is not sensitive enough to determine whether the proteins are indeed physically interacting in the cytoplasm.

As reviewer's suggested, the immunofluorescence imaging can only indicate their overlaps in cellular distributions, while their physical interaction was determined by various co-immunoprecipitations that reveal their complexes at the endogenous level or upon co-transfection (Figs. 3G-3J). Interaction between PPM1A-YAP was also supported by the in vitro phosphatase assays (Fig. 4B-4C).

13. I fail to see a significant difference between controls and PPM1A-expressing cells in Fig. 4F.

We quantified the fluorescence intensity of YAP and PRP4K by ImageJ software. A substantial amount of YAP was distributed outside of the nucleus (DAPI/blue) in the presence of PRP4K, which was entirely resided into the nucleus when PPM1A was coexpressed (Fig. 4I, S4D).

14. Some of the critical experiments should be performed in the absence of Mg2+ to enhance the argument of the authors that it is indeed PPM1A that is involved in the regulation of YAP.

Thanks for the reviewer's suggestion. We performed the in vitro phosphatase assay in the presence of EDTA that deprived the Mg^{2+}/Mn^{2+} from PPM1A, which prevented the dephosphorylation of pYAP (Fig. 4B, S4B). This observation, in addition to using the phosphatase-dead PPM1A in various experimental settings, suggests that PPM1A is responsible for YAP dephosphorylation.

15. Why and how does PPM1A target several residues in YAP? Is there a specific

consensus for PPM1A and do the YAP sites that seem to be dephosphorylated by PPM1A fit that consensus sequence?

To our knowledge, no specific consensus of PPM1A has been suggested up to date and it is very common that PPM1A attacks and dephosphorylates several phospho-residues on its substrate. In addition, what we observed during in vitro phosphatase assay that PPM1A robustly dephosphorylated a YAP peptide spanning the S127 residue (Fig. 4C), suggests that YAP is a fitting substrate of PPM1A.

16. The number of animals used in the DSS experiments seems low, considering that this procedure is known to lead to experimental variability.

Per the reviewer's request, we performed a new set of experiments for DSS-induced colitis in mice, which consolidated our previous observations (Fig. 6A-6C and Fig. S6C-6D). Notably, we have now collected the data for genetic rescue of colitis phenotypes in Ppm1a$^{-/-}$ mice by crossing the Lats1$^{+/-}$ heterozygotes. The severe symptoms of colitis in Ppm1a$^{-/-}$ mice were nicely rescued by the deficiency of LATS1, which also substantially reduced the pYAP level in PPM1A-KO colons (Fig. 6H-6I and Fig. S6E-S6G).

.

17. How does the data on TGF-beta shown in Fig. 7D correlate with the fact that previous studies have shown that TGF-beta can influence regeneration in the liver?

According to literature, it appears that the effects of TGF-beta on liver regeneration are sensitive to and critically depends on the stages and contexts of liver [4]. In the liver regeneration conditions that we were employing, we did observe that SB431542 somehow reduced the liver cell proliferation (at 48 h post hepatectomy) and it appeared unrelated to PPM1A. These observations could result from the complex regulations of TGF-beta signaling, or by some non-specific effects of drug, which both we believe are beyond the scope of this study.

Minor points:

1. Manuscript should be revised in terms of grammar for enhanced understanding by the readership.

Thank you for your suggestion. We have now carefully revised the sentences and descriptions, and believe this revision can help us to better convey our scientific conclusions to readers.

2. Line 79: Typo in Mats.

Thanks, and the text has been revised.

3. Line 111: Recent publications revealing the importance of STRIPAK in the

regulation of mammalian Hippo signalling should be included as references.

Thank you for your suggestion. The article has been cited in the Introduction now.

4. Line 146: Typo in MAP4K1

Thank you for your carefulness, the text has been revised now.

5. Fig. S2E should include PPM1A WB data showing levels of PPM1A in the different genotypes.

Thanks, and we have added the immunoblotting result of PPM1A in new Fig. S2G.

6. Analysis of YAP sub-cellular localization in organoids is not entirely clear.

Per the reviewer's suggestion, we repeated the analyses of YAP localization in organoids, which have improved the quality of imaging, indicating that PPM1A deficiency decreased the cells with YAP in the nucleus, even among the Edu-positive group (Fig. 5F).

7. Fig. 7B: Typo in regeneration. Missing information next to images of livers (presumably statistical analysis?).

Thank you for your suggestion. The clerical error in the picture has been modified. In addition, we repeated the liver regeneration experiment according to the reviewer 4's suggestion, and replaced the original picture with a clearer one (Fig. 7B).

Rev. 2:
This manuscript from Zhou et al revealed that PPM1A phosphatase activity positively regulates mammalian liver and intestinal regeneration by targeting its newly identified substrate YAP. The authors employed an "unbiased phosphatome screening" consisting of cDNA of 40 known serine/threonine phosphatases to kick-start their investigation via YAP dependent transcriptional activity as a readout. Along with other reported phosphatases, the author identified PPM1A regulate the phosphorylation status of YAP at crucial Ser127 (as well as Ser109 and Ser366) thereby translocating YAP to the nucleus for YAP-TEAD transcription. In addition, PPM1A can also regulate phosphorylation status of YAP in the nucleus. The authors further provided evidences of PPM1A physical interactions with YAP using confocal microscopy and co-immunoprecipitation. Finally, the authors showed that PPM1A phosphatase activity was indeed physiologically important for YAP-TEAD mediated downstream effects. PPM1A phosphatase activity dictates YAP activation and nuclear localization in both cell and animal models, and absence of PPM1A revealed expected disruption in YAP-TEAD dependent organ regeneration and repair, the impairment

could be rescued by inhibiting kinase activity of MST1/2.

It is a well conducted study on the elucidation of involvement and roles of PPM1A in Hippo-YAP signalling. With the questions below addressed, I feel that it is worth publishing in PLOS Biology.

1. In the abstract (line 48-49) and the highlight section (line 73-74), the authors stated that phenotypes of PPM1A KO mice inability in organ regeneration was rescued by prevention of PPM1A-YAP regulation. If I were to understand correctly, only allowing PPM1A to regulate YAP will then allow normal regeneration of injured organ. How can prevention of PPM1A-YAP regulation rescued the regeneration?

Thanks for the reviewer's positive comments and suggestions. We agreed with the reviewer that the original description is very confusing, although our intention is to suggest that prevention of the PPM1A-YAP regulation, such as through the introduction of YAP 2SA mutant, can rescue phenotypes of PPM1A-KO cells and organoids. We have now revised the manuscript to remove this confusing point. In addition to pharmacological inhibition, we added in the revised manuscript the new data for a genetic rescue for colitis phenotypes in Ppm1a$^{-/-}$ mice by deficiency of LATS1. The breeding started last year but was delayed by the CoV-19 pandemic. The severe symptoms of colitis in Ppm1a$^{-/-}$ mice were nicely rescued by LATS1 heterozygote (Fig. 6H-6I and Fig. S6E-S6G). These observations further strengthened our opinion that PPM1A regulated organ regeneration via regulation of the Hippo-YAP mechanism.

2. Can the author comment how may PPM1A be localized (cytosol or nucleus) in basal condition? From the data available in the text, PPM1A seems to follow YAP localization? As the author mentioned in the main text, HA-YAP overexpressed cell model resulted in PPM1A mainly nuclear localization while if HA-LATS1 is overexpressed, PPM1A can exist substantially in the cytosol. Perhaps PPM1A localized based on YAP phosphorylation status? How much a role can PPM1A act as a phospho-YAP sensor?

Thanks for the reviewer's insightful suggestion. We observed that PPM1A was mainly located in the nucleus in the resting state in most types of cell [1] (Figs. 1I, 1J, 2A, 3F), and a significant subset of them was exit to the cytoplasm when the Hippo signaling was activated (Fig. 3F). Intriguingly, LATS1, but not LATS2 (data not shown), robustly drove the cytoplasmic distribution of PPM1A (Fig. 3D-3E). The underlying mechanism for this signaling-induced cellular redistribution of PPM1A was still unknown, as well as the basis for the distinct effect between LATS1 and LATS2. Because both LATS kinases can phosphorylate YAP, we speculate that LATS1-induced modification of PPM1A or transporter(s) of PPM1A, but not the phospho-YAP, lead to the cytoplasmic residence of PPM1A. However, it is also possible that a subtle difference in YAP phosphorylation mediated by LATS1 and LATS2, resulting in a distinct PPM1A

interaction. At current stage of the data, I believe that we cannot define PPM1A as a sensor of phospho-YAP, but we can propose that PPM1A functions as a guardian of YAP/TAZ for preventing its being phosphorylated and degraded.

3) Edits: The author should standardize the annotation of nucleus marker "DAPI" or "DAP1" in the figures.

Thanks, and we have now corrected these errors.

Rev. 3:
The Hippo pathway is an essential organ size and homeostatic pathway. The authors investigated the mechanism for Yap/Taz dephosphorylation and discovered that PPM1A/PP2Cα as an important p'ase to counter the Hippo mediated phosphorylation of Yap/Taz. Overall the data are convincing and this is an important advance for the Hippo field. There are many areas that can be improved in the writing and also some data presentation:

Comments:
1. English usage should be carefully re-evaluated throughout the manuscript. In some places, such as abstract, the manuscript is confusingly written.

Thanks for the reviewer's positive comments and suggestions. We have accordingly revised the manuscript carefully to better convey our views.

2. This statement regarding Figure 2B should be clarified as it is unclear if it is accurate: "Intriguingly, the extent of YAP phosphorylation in resting state was comparable to those YAPs under glucose starvation/energy stress."

Thanks and we agreed with the reviewer on this point. We rewrote this sentence to have a clearer description.

3. Please state the phenotype of the PPM1A mutant mice early in the manuscript rather than at the end. If this is the main pase for Yap/Taz it would be predicted to be embryonic lethal.

Thanks for the reviewer's suggestion. We added the data in the Results section (Fig. 5A-B) along with a brief statement. In addition, please also refer to the 3rd question of reviewer 1. We used double-depletion of PPM1A and PPM1B to confirm a redundant effect between these two closely-related phosphatases in regulation of Hippo-YAP signaling (Fig. 5A-5B). On the other hand, the Ppm1b$^{d/d}$ gene-trap mice, as we have reported previously [3], are viable and born with a normal Mendelian distribution. However, no offspring of genotype Ppm1b$^{d/d}$/Ppm1a$^{-/-}$ can be successfully obtained, supporting our hypothesis that PPM1B may compensate for PPM1A during embryonic development in Ppm1a$^{-/-}$ mice.

4. Figure 3C is an important data point and should be quantified to make the point more conclusively.

Thanks. We have quantified Fig 3C, which revealed that PPM1A dephosphorylated the pYAP (S127) that was induced both by the WT LATS1 and its phosphomimetic form.

5. For subcellular localization of endogenous PPM1A there should be a control such as siRNA against PPM1A (Fig 3F).

Thank you for your suggestion. We used the CRISPR-based PPM1A KO cells in Fig. 2A to confirm that the anti-PPM1A antibody used in immunofluorescence detection of endogenous PPM1A was specific.

6. The interaction data at end of Figure 3 would be strengthened by looking at endogenous proteins if possible.

Per the reviewer's suggestion, we conducted an endogenous co-IP experiment in the liver lysates obtained from mice upon 2-day post hepatectomy, and an obvious interaction between the endogenous PPM1A and YAP was detected (Fig 3H).

7. Data in Fig 4 A, B are convincing and support the hypothesis.

Thanks. Per the request of the reviewer 1, we further validated observations of the in vitro phosphatase assays with a recombinant PPM1A from the bacteria, and a recombinant phosphatase domain of PPM1A together with the synthesized YAP peptide (Fig. 4B-4C) for the measurement of the K_{cat}/K_m *coefficient.*

8. Data in figure 5D (organoids) needs to be improved - current version is poor quality.

Thanks for the suggestion. We repeated the analysis of YAP localization in organoids, which had improved quality of imaging and indicated that PPM1A deficiency decreased the number of cells with nucleo-YAP, even among the Edu-positive group (Fig. 5F).

9. Data in Fig 6 F can also be improved.

Thanks, and we have attempted to improve the quality of this figure (Fig. 6F).

Rev. 4:
In this manuscript PBIOLOGY-D-20-01910R1 entitled 'PPM1A dephosphorylates and activates YAP to govern mammalian intestinal and liver regeneration', Zhou et al.

presented an impressive amount of biochemical data to prove that protein phosphatase magnesium dependent 1A (PPM1A), a member of PP2C subfamily phosphatases, was a direct modifier of YAP. Accordingly, genetic ablation and depletion of PPM1A resulted in YAP/TAZ cytoplasmic retention, while PPM1A deficiency in organoids and mice resulted in reduced nucleo-YAP and diminished cell proliferation, which leading to severe regeneration defects in gut epithelium during colitis and in livers upon injury. Although these findings were potentially interesting, the authors should address the following concerns.

Main concerns:
1. Control is missing in Figure 1G, 2D, 2K-2L, S2C-S2D, and 3A which the mRNA levels of CTGF and CYR61 in corresponding YAP overexpression or knockdown/knockout are expected to be tested for their expression.

Thanks for the reviewer's overall positive comments and suggestions. CTGF and CYR61 are two well-defined target genes of the YAP/TAZ-TEADs transcription complex [5]. Per the reviewer's request, we added the corresponding control experiment where YAP was depleted in HEK293 cells, which downregulated the mRNA levels of CTGF and CYR61 significantly (Fig. S1D). In addition, mRNA levels of CTGF and CYR61 were significantly downregulated upon shRNA-mediated depletion of YAP (Fig. 4F, S4C) but were upregulated by deletion of MST and LATS kinases (Fig. 3A), as expected.

2. How to explain that PPM1A is mainly located in the nucleus while the pYAP is usually retained in the cytoplasm? Figure 1H and 3C, lower bands were supposed to be endogenous p-YAP(S127) However, endogenous p-Yap was not altered by PPM1A overexpression in cells. Please address these inconsistence.

We have intriguing observations that PPM1A was induced to translocate to the cytoplasm, upon activation of Hippo signaling and possibly mediated by LATS1 (Fig. 3D-3F). The regulation partially explained the dramatic effects of PPM1A to YAP activation, as evidenced by various gain- or loss-of functions. We agree with the reviewer that the lower band under pYAP (S127) might be the endogenous pYAP, though not validated. However, it is reasonable that most endogenous pYAP was unaffected by transfection of PPM1A, as only a small subset of cells were being transfected when using the PEI-mediated transfection (Fig. 1F, 3C).

3. The shift Phos-Tag bands have a different trend in PPM1A knockout cells in Figure 2B, why? The author should add LATS1 as control in Figure 3B; Why LATS1 S909D and T1079D caused different changes in pYAP in Figure 3C?

YAP/TAZ proteins are excessively modified either in resting state or upon Hippo signaling and these modifications interact each other. However, PPM1A is only responsible for dephosphorylation of YAP/TAZ only on several residues. Therefore, we

believe it is not surprising to measure a somewhat different status of YAP phosphorylation between PPM1A deletion and glucose starvation-caused alteration, under phos-tag electrophoresis (Fig. 2B). Per the reviewer's suggestion, we added the LATS1 WT as the control (Fig. 3B). In addition, we found that LATS1 phosphomimetic T1079 was active and drove the downstream YAP phosphorylation, but LATS1 S909D not, similar to previous report [6] (Fig 3C).

4. In Figure 2J, depletion of PPM1A in HEK293 significantly decreased TEAD-driven activity both in resting state and under serum starvation. However, in Figure 2C, depletion of PPM1A in HEK293 attenuated TEAD-driven activity only in resting state but not serum starvation state, why?

HEK293A cells respond robustly to Hippo-YAP signaling. As a result, the levels of TEAD-driven transactivation were so low to show a significant difference when PPM1A was further deleted, a so-called "floor effect".

5. Figure 2B, the levels of p-YAP in resting state was comparable to those under glucose starvation/energy stress. However, in previous study, glucose starvation significantly increased YAP phosphorylation. (1. AMPK modulates Hippo pathway activity to regulate energy homeostasis. 2. Cellular energy stress induces AMPK-mediated regulation of YAP and the Hippo pathway). The authors should address these inconsistent findings.

Thanks. We believe that there is a rapid increase of phospho-YAP upon glucose starvation in the Fig. 2B, observable but not so obvious. Because Hippo-YAP signaling is particularly sensitive to cell status, we repeated this experiment with an optimized cell density and treatments, which revealed an apparent increase of pYAP (S127) upon glucose deficiency, or under the PPM1A deletion (Fig. S2A). The glucose starvation-induced phosphorylation of YAP is consistent with previous observations [7-9].

6. If PPM1A is mainly responsible for directly eliminating phospho-S127 on YAP, why does PPM1A interact with LATS1? (Figure 3I)? Are PPM1A and LATS1 competitively combined with pYAP/YAP?

PPM1A was mainly resided in the nucleus but was driven to the cytoplasm by the activated LATS1, where it co-localized with YAP and LATS1 (Fig. 3D-3F) and dephosphorylated YAP to stabilize and maintain its activity. We believe that it is a typical negative feedback loop of Hippo-YAP signaling, to prevent the overquick and excessive inactivation of YAP/TAZ. Because PPM1A was recruited by LATS1, we did not focus on their mutually competitive regulation. We speculate that they may form a complex containing both kinases and phosphatases and other components, alike to STRIPAK.

7. Why is XMU-MP-1 not used in figures 5G-H and 7D, but in figure 6? The author should add the phenotype of XMU-MP-1 treatment in the corresponding experiments.

We have indeed attempted to treat XMU-MP-1 in intestinal organoids. However, these organoids were toxic to XMU-MP-1, alike to many other in vitro cultured organoids that were sensitive to drugs. This fact prevented us to perform the suggested experiment. Per the reviewer's request, we have applied XMU-MP-1 into mice with hepatectomy surgery, which reduced the pYAP levels and nicely restored the proliferation potential of hepatocytes in PPM1A KO mice (Fig. 7F-7G).

8. The lower panel in Figure 7B needs to be replaced with a cleaner one.

Thanks for the reviewer's suggestion. We repeated the experiment of liver regeneration post hepatectomy to address the above questions, and a clearer liver picture was obtained and replaced the previous one (Fig. 7B).

References:

1. Xiang, W., et al., *PPM1A silences cytosolic RNA sensing and antiviral defense through direct dephosphorylation of MAVS and TBK1.* Sci Adv, 2016. **2**(7): p. e1501889.
2. Sasaki, M., et al., *Disruption of the mouse protein Ser/Thr phosphatase 2Cbeta gene leads to early pre-implantation lethality.* Mech Dev, 2007. **124**(6): p. 489-99.
3. Chen, W., et al., *Ppm1b negatively regulates necroptosis through dephosphorylating Rip3.* Nat Cell Biol, 2015. **17**(4): p. 434-44.
4. Oe, S., et al., *Intact signaling by transforming growth factor beta is not required for termination of liver regeneration in mice.* Hepatology, 2004. **40**(5): p. 1098-105.
5. Zhao, B., et al., *Inactivation of YAP oncoprotein by the Hippo pathway is involved in cell contact inhibition and tissue growth control.* Genes Dev, 2007. **21**(21): p. 2747-61.
6. Chan, E.H., et al., *The Ste20-like kinase Mst2 activates the human large tumor suppressor kinase Lats1.* Oncogene, 2005. **24**(12): p. 2076-86.
7. DeRan, M., et al., *Energy stress regulates hippo-YAP signaling involving AMPK-mediated regulation of angiomotin-like 1 protein.* Cell Rep, 2014. **9**(2): p. 495-503.
8. Wang, W., et al., *AMPK modulates Hippo pathway activity to regulate energy homeostasis.* Nat Cell Biol, 2015. **17**(4): p. 490-9.
9. Mo, J.S., et al., *Cellular energy stress induces AMPK-mediated regulation of YAP and the Hippo pathway.* Nat Cell Biol, 2015. **17**(4): p. 500-10.
