# Supplementary material for: The protein phosphatase PPM1A dephosphorylates and activates YAP to govern mammalian intestinal and liver regeneration
Source: PLoS Biol. 2021 Feb 25;19(2):e3001122. doi: 10.1371/journal.pbio.3001122 (PMC7978383; doi:10.1371/journal.pbio.3001122)
Supplement: Attachment — Submitted filename: Response to the reviewers.pdf [file pbio.3001122.s012.pdf]

<u>Response to the reviewers' comments:</u>

Reviewers' comments:

Rev. 1:

The revised version of the manuscript by Zhou et al. is a markedly improved report that more convincingly shows that PPM1A is involved in the regulation of YAP. Importantly, the authors addressed appropriately the major points that required improvement before the manuscript could be recommended for publication in PLoS Biol.

In particular, the fact that the authors now show that recombinant PPM1A can dephosphorylate YAP in vitro suggests that, indeed, PPM1A is acting directly on YAP. Moreover, as suggested by the reviewers, the authors also provide evidence that, as predicted by their model and the proposed mechanism of action of PPM1A, a non-phosphorylatable form of YAP is largely refractory to the action of PPM1A. The authors also provide evidence that supports their claim that PPM1B could compensate for PPM1A loss in the regulation of YAP. Although this is not entirely resolved given the inability of testing this adequately in vivo, the in vitro results showing an enhancement of YAP phosphorylation when both phosphatases are depleted, suggests this compensatory mechanism is possible. The fact that the in vivo experiments have been complemented by the use of a larger number of animals also gives strength to the authors' claims that PPM1A is involved in regeneration in vivo.

In conclusion, the authors have provided a sufficiently enhanced manuscript, which is suitable for publication.

Thanks for the positive comments and approval of the revision.

Rev. 4:

My concerns have been addressed satisfactorily.

Thanks for the positive comments and approval of the revision.
